# Low-dose memantine attenuated methadone dose in opioid-dependent patients: a 12-week double-blind randomized controlled trial

**DOI:** 10.1038/srep10140

**Published:** 2015-05-19

**Authors:** Sheng-Yu Lee, Shiou-Lan Chen, Yun-Hsuan Chang, Po See Chen, San-Yuan Huang, Nian-Sheng Tzeng, Liang-Jen Wang, I Hui Lee, Tzu-Yun Wang, Kao Chin Chen, Yen Kuang Yang, Jau-Shyong Hong, Ru-Band Lu

**Affiliations:** 1 Department of Psychiatry, Kaohsiung Veterans General Hospital, Kaohsiung, Taiwan; 2 Department of Psychiatry, College of Medicine and Hospital, National Cheng Kung University, Tainan, Taiwan; 3 Department of Neurology, School of Medicine, Kaohsiung Medical University, Kaohsiung, Taiwan; 4 Institute of Allied Health Sciences; 5 Department of Psychiatry, Tri-Service General Hospital, National Defense Medical Center, Taipei, Taiwan; 6 Department of Child and Adolescent Psychiatry, Kaohsiung Chang Gung Memorial Hospital and Chang Gung University College of Medicine, Kaohsiung, Taiwan; 7 Laboratory of Neurobiology, NIH/NIEHS, Research Triangle Park, NC, USA; 8 Institute of Behavioral Medicine, College of Medicine and Hospital; 9 Addiction Research Center, National Cheng Kung University, Tainan, Taiwan; 10 Center for Neuropsychiatric Research, National Health Research Institutes, Miaoli, Taiwan

## Abstract

Low-dose memantine might have anti-inflammatory and neurotrophic effects mechanistically remote from an NMDA receptor. We investigated whether add-on memantine reduced cytokine levels and benefitted patients with opioid dependence undergoing methadone maintenance therapy (MMT) in a randomized, double-blind, controlled 12-week study. Patients were randomly assigned to a group: Memantine (5 mg/day) (n = 53) or Placebo (n = 75). The methadone dose required and retention in treatment were monitored. Plasma tumor necrosis factor (TNF)-α, C-reactive protein (CRP), interleukin (IL)-6, IL-8, transforming growth factor (TGF)-β1, and brain-derived neurotrophic factor (BDNF) levels were examined during weeks 0, 1, 4, 8, and 12. General linear mixed models were used to examine therapeutic effect. After 12 weeks, Memantine-group required a somewhat lower methadone dose than did Placebo-group (P = 0.039). They also had significantly lower plasma TNF-α and significantly higher TGF-β1 levels. We provide evidence of the benefit of add-on memantine in opioid dependent patients undergoing MMT.

Opioid dependence is often characterized by repetitive drug-seeking and drug-taking behaviors with severe public health consequences. Current efforts to taper individuals off opioids often lead to limited results because of a high relapse rate and troublesome subjective symptoms. Although methadone maintenance therapy (MMT) has been suggested as effective for opioid dependence^1^, after methadone is discontinued, the opioid dependents often relapse. Using MMT alone may not be sufficient for treating opioid dependence. Therefore, there is a need to develop adjuvant therapeutic interventions for opioid-dependent patients during long-term MMT.

Opioids cause oxidative stress and inflammatory responses. Evidence from human and animal studies *in vivo* and *in vitro* suggest that opioid abuse may have adverse immunomodulatory effects on innate and adaptive immune responses^2^. *In vitro* studies report that acute morphine treatment alters the production of various cytokines, including tumor necrosis factor (TNF)-α and IL-6^3^,^4^. In the brains of opioid-dependent patients, higher cytokine expression levels were detected in noradrenergic locus coeruleus cells^5^. Chronic heroin use has been associated with decreased serum concentrations of nerve growth factor and brain-derived neurotrophic factor (BDNF)^6^. Another study showed that the increase of BDNF in the nucleus accumbens was closely related to dependence on cocaine and other drugs, and to dependency relapse^7^. In addition, BDNF is involved in long-term behavioral adaptation induced by drug dependence^8^.

Current treatment for opioid dependence in practice remains less than ideal. Although agonist maintenance using methadone or buprenorphine remains the treatment of choice^9^,^10^, using these agonists to treat young people, newly diagnosed patients, or abusers of prescribed opioids remains controversial. MMT is sometimes not available or acceptable to many patients, nor is it universally effective. Because inflammation and neurodegeneration have been reported in the progression of opioid dependence^11^, treatment combining anti-inflammatory and neuprotective agents may provide more benefit than current management without these agents. We recently showed that using low-dose (0.02 mg/kg) memantine, an N-methyl-D-aspartate (NMDA) receptor antagonist, abolished morphine-induced conditioned-place-preference behavior in rats because of its IL-6-modulating effect in the medial prefrontal cortex^12^. Our preliminary clinical data also showed that low-dose memantine added to valproate given to patients with bipolar II disorder significantly attenuated plasma cytokines^13^. We hypothesized that a low dose of add-on memantine would therapeutically benefit opioid-dependent patients. We conducted a double-blind, placebo-controlled study of add-on low-dose memantine (5 mg/day) in opioid-dependent patients undergoing MMT to evaluate whether memantine would reduce the dose of methadone needed.

## Methods

### Study design

The research protocol was approved by the Institutional Review Board for the Protection of Human Subjects at National Cheng Kung University Hospital, and the methods were carried out in accordance with the approved guidelines. After the study had been completely described to the participants, they all signed written informed consent forms.

Opioid-dependent patients were recruited from the MMT program. None of the patients received methadone prior to this trial; all patients were newly inducted onto methadone. Each participant was initially interviewed and evaluated by an attending psychiatrist, and then the evaluation was confirmed by a research team member well-trained and experienced in using the Diagnostic and Statistical Manual of Mental Disorders, fourth edition (DSM-IV) criteria and the Chinese Version of the Mini International Neuropsychiatric Interview (MINI)^14^. We chose the MINI to evaluate heroin-dependent patients because it is difficult for them to complete 4 to 6 hours of a structured interview, such as the Chinese Version of the Modified Schedule of Affective Disorder and Schizophrenia-Lifetime (SADS-L)^15^. Inclusion criteria were men and women between 18 and 65 years old who met the DSM-IV criteria for current opioid dependence and who used opioids daily. Exclusion criteria were a major or minor mental illness other than opioid dependency, antisocial personality disorder, and cognitive disorders. Other exclusion criteria were being pregnant or nursing an infant, having taken any anti-inflammatory medications within 1 week before the study, or having a history of one or more uncontrolled major physical conditions such as chronic diabetes mellitus or chronic hypertension.

### Procedures

Participants were randomly assigned to one of two groups: Placebo (taking methadone + one daily placebo capsule) or Memantine (taking methadone + one daily 5-mg memantine sustained-release capsule) for 12 weeks. The randomization strategy for treatment was simple randomization using excel’s random number generator. Methadone maintenance treatment was launched in Taiwan by the government in 2006 and made available countrywide for treatment of opioid dependence in Taiwan since 2007. The guideline in Taiwan from Department of Health suggested an initial dosage range from 10–40 mg per day, but preferably not over 30 mg/day. An increase or decreased by 5 mg increment for dosage adjustment was also suggested by the guideline. The adjustment of dosage of methadone was made according to clinician’s evaluation, patients’ subjective responses including withdrawal symptoms and tolerance to methadone. The primary outcome of the study was to compare the methadone dose required, retention rates, and concomitant opioid use of participants in the 12-week trial. The methadone doses required were recorded at baseline and on day 7 of weeks 1, 4, 8, and 12. The secondary outcome of the study was to compare the immunological parameters including TNF-α, CRP, IL-6, IL-8, and TGF-β1 levels and plasma BDNF levels at baseline and on day 7 of weeks 1, 4, 8, and 12. All patients received evaluation of sections of Opiate Treatment Index (OTI) including drug use, criminality, and health at endpoint as behavioral measures and side effect checklist for adverse event.

### Outcome Measurement

The dose used in the current study was determined from animal and human studies of addiction behavior. In an animal study^12^, we found that using a low dose of memantine (0.2–1 mg/kg/day) abolished morphine-induced conditioned place preference behavior in rats because of its anti-inflammatory and neurotrophic effects in the addiction-related brain area. We converted that to a human-equivalent dose (0.03–0.16 mg/kg) (U.S. Department of Health and Human Services, 2005). By assuming the average human weight to be 60 kg, we determined that the median daily dose for humans is 5 mg (range: 1.8–9.6 mg/day). We have also used add-on oral memantine (5 mg/day) with valproic acid (VPA) to treat patients with bipolar II disorder. We found that although add-on memantine + VPA may not be more effective for clinical symptoms than was placebo + VPA, memantine may have improved plasma TNF-α levels, but that it had little effect on other cytokines^13^. We therefore used this dose to treat heroin-dependent patients.

Ten milliliters of whole blood was withdrawn from the antecubital vein of each patient at baseline and on day 7 of weeks 1, 4, 8, and 12. Plasma, which was isolated from the whole blood after it had been centrifuged at 3000 *g* for 15 min at 4 °C, was immediately stored at −80 °C. Cytokine levels were quantified using an antibody pair assay system (Flexia; BioSource Intl., Camarillo, CA). A BDNF kit (Quantikine Human BDNF kit; R&D Systems, Minneapolis, MN) and an enzyme-linked immunosorbent assay (ELISA) reader (SpectraMax-M2; Molecular Devices, Sunnyvale, CA) were used to analyze the plasma BDNF level. Samples were processed and data analyzed according to the manufacturer’s instructions. The immunological parameters (TNF-α, CRP, IL-6, IL-8, and TGF-β1) and BDNF were assessed.

### Statistical Analyses

The demographic and clinical characteristics of the patients and their baseline methadone dose, cytokine levels, and BDNF levels were compared between groups using one-way analysis of variance (ANOVA) for continuous variables and χ^2^ tests for categorical variables. The randomization strategy for treatment was simple randomization using excel’s random number generator. Data are means ± standard deviation (SD). Arithmetic transformations were used to produce approximately normal distributions for further analysis; log (*x *+ 1) was used for cytokine levels. Potential prognostic factors included the treatment duration (0–12 weeks), memantine dose, gender, and age. Because there were repeated assessments, mixed-effect-model analysis was used to control for time effects, age, and gender, and used on longitudinal outcomes (methadone doses, cytokine levels, and BDNF levels) to evaluate the possible effects of the prognostic factors on the response values. We used mixed-effect models to analyze the effect of add-on placebo and memantine during 12 weeks treatment in opioid-dependent participants. A total of 8 models ran with each outcome as a dependent variable. In each model, treatment received (memantine vs. controls), treatment course, treatment received X treatment course, gender, and age were included as independent variables. The covariance structure employed was compound symmetry model. The placebo group was used as reference group. The interaction term of treatment received and treatment duration was regarded as effect of add-on memantine. The retention rate was estimated using the Kaplan-Meier product limit estimate method, and survival curves for the two groups were compared using the Wilcoxon rank sum test. SPSS 18.0 for Windows was used for statistical computations. Significance was set at p < 0.05.

## Results

### Study Participants

**Figure 1:**
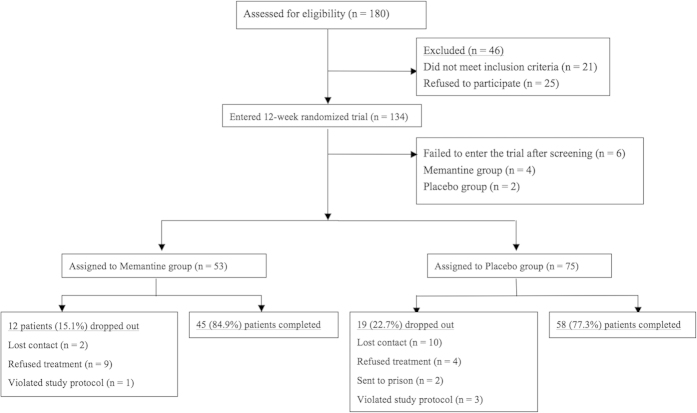
CONSORT diagram showing the disposition of patients in the study.

One hundred eighty opioid-dependent patients were screened for eligibility (See Fig. 1 for the CONSORT Flow Diagram). Forty-six of those screened declined to participate (failed to complete the evaluation and were not interested in treatment other than methadone). Finally, 134 opioid-dependent participants entered the study and underwent randomization. owever, 4 patients in the Memantine group and 2 patients in the Placebo group failed to enter the trial after screening. The rest patients were randomly assigned to the Memantine group (n = 53) or the Placebo group (n = 75) for 12 weeks. One hundred three (80.5%) of the 128 patients completed the double-blind phase, and 25 (19.5%) dropped out (Placebo: n = 17; 22.7%; Memantine: n = 8; 15.1%). Their reasons for discontinuing the study were as follows: Loss of follow-up for an unknown reason (Placebo: n = 10; Memantine: n = 2), refused treatment (Placebo: n = 2; Memantine: n = 5), violation of protocol (Placebo: n = 3; Memantine: n = 1), and incarcerated in prison during treatment (Placebo: n = 2; Memantine: n = 0). No adverse events were reported in either treatment group.

### Primary and Secondary Outcomes

The demographic and clinical characteristics, baseline methadone dose scores, and BDNF and cytokine levels of the patients were similar in both patient groups at baseline, but all cytokine levels were distributed erratically and showed a significant level of positive skew (Table 1). There were no significant behavioral differences in drug use, criminality nor physical health in OTI between the Memantine and Placebo groups before or after 12 weeks treatment (Table 1). Past amount of heroin use was not recorded because the purity of heroin varies widely. None of the patients received methadone or other opioid agonists prior to this trial; all patients were newly inducted onto methadone since methadone was launched in Taiwan by the government in 2006 and made available countrywide for treatment of opioid dependence in Taiwan since 2007.

**Figure 2:**
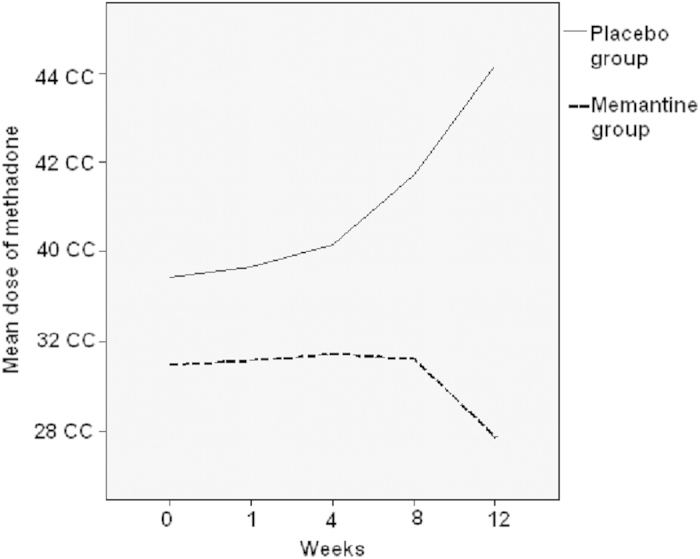
Change in the mean dose of oral methadone in the Memantine and Placebo groups after 12 weeks of treatment.

**Figure 3:**
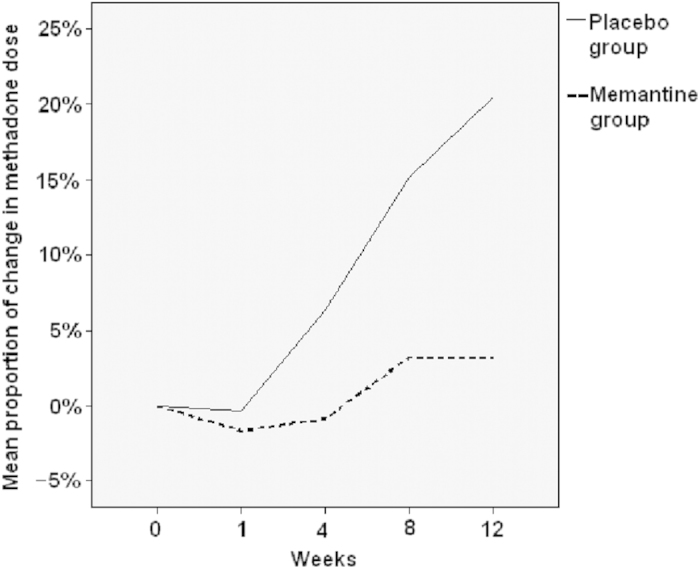
The mean proportion of change in the oral dose of methadone, normalized using the baseline data (week 0 = 100%) of each patient in the Memantine and Placebo groups after 12 weeks of treatment.

The data presented in Table 2 was the main effect (treatment received, treatment course, gender, and age) and interaction term (treatment received X treatment course) from each model (using each outcome as dependent variable). The change in the required methadone dose and its normalization against the baseline dose (week 0 = 100%) after 12 weeks of treatment in the two groups are plotted in Figs. 2,3. The required methadone dose in the Memantine group was significantly lower both before (p = 0.034) and after normalization (p = 0.025) (Figs. 1,2; Table 2). Our finding suggests that for those treated with 5 milligram of memantine, the required methadone dose decreased by 0.948 mg over each visit compared to Placebo group when controlling for the effects of gender and age The Memantine group had a significantly lower TNF-α level (p = 0.004) and a significantly higher TGF-β level (p = 0.017) than did the Placebo group, but BDNF and other cytokine levels were not significantly different. *There are also several significant main effects of age, gender or treatment course in the models analyzed in Table 2. However, these significant model main effects do not affect the outcome or do not represent effect of add-on memantine*. If we control for baseline methadone dose, the required methadone dose decreased by 0.950 mg over each visit (P = 0.032) compared to the placebo group when controlling for the effects of gender and age.

There were no significant differences in retention rates between the Memantine and Placebo groups (Table 3). As for adverse effect, no significant differences were found between the Memantine and Placebo groups (Table 4). However, marginally more adverse symptoms in urogenital system were found in Memantine group compared to Placebo group (P = 0.07).

## Discussion

There are no published reports that both evaluate add-on low-dose memantine used to treat opioid-dependent patients undergoing MMT and closely monitor patient cytokine levels during treatment. We found that add-on memantine was significantly more effective than placebo for decreasing dose of methadone required. We also found that add-on memantine plus methadone was significantly more effective than methadone alone for decreasing TNF-α levels and increasing TGF-β levels, but not for modulating CRP, IL-6, IL-8, or BDNF levels for opioid-dependent patients. However, the difference in increase of the TGF-β levels appears to be because the levels in Placebo group dropped but not increase in Memantine group. Although no significant differences in side effect was found between Memantine and Placebo groups, marginally more adverse symptoms in urogenital system were found in Memantine group (P = 0.07). Our study provides initial evidence that memantine may be effective for reducing the patient’s methadone dose required and decreasing some inflammatory factors in opioid-dependent patients.

The main finding of the current study, difference in methadone dosage, was only significant after considering effect of time using mixed model analysis. At endpoint, only borderline difference in methadone dosage between mematine and control groups was seen (Table 1). In other words, current study demonstrated the effect of memantine compared to placebo as 1 mg vs. 8 mg increase in methadone dose required. Methadone maintenance treatment was launched in Taiwan by the government in 2006 and made available countrywide in 2007. One recent study comprised of a cohort of 33,549 patients recruited from 2006 to 2008 reported that over half over patients received methadone less than 45 mg per day and the mean dose was 46.5 ± 20.9 mg/day^16^, while average treatment duration was 171.5 days. The average dose reported by Liao *et al*.^16^ is similar to the endpoint dose in the Placebo group in the current study. On the other hand, the guideline in Taiwan suggests an increment of 5 mg of methadone a day for dose adjustment. Therefore, our finding suggests that add-on memantine may decrease methadone dose needed by about 2 increment levels compared to national average dosage. However, lacking of any behavioral or craving or self-administration data, the clinical relevance of our finding still requires further study. Furthermore, due to lower average dose and shorter clinical experience of methadone in Taiwan compared to that in the U.S.^17^,^18^, the result of the current study may not be applicable to other countries or ethnics.

Heroin-dependent patients may develop a tolerance for methadone. However, we found that, after 12 weeks of MMT plus add-on low-dose memantine, the dose of methadone required by Memantine-group patients was significantly lower than the dose required by Placebo-group patients. Because the retention rate (which indicates a craving for heroin) did not differ between the two study groups, our results support the notion that a reduction in the required dose of methadone indicates that the patient’s tolerance to methadone has been inhibited and that the patient’s symptoms when withdrawing from heroin will be less severe. In clinical practice, patients who abuse opioids may continue MMT for years. Although methadone tolerance has not yet been reported in Han Chinese people, it is an important issue that needs to be addressed and managed. In addition, methadone is neurotoxic: chronic methadone use might damage the striatal dopamine transporter in humans^19^ and impair cognitive function and sustained attention^20^,^21^. Thus, patients undergoing long-term MMT might experience more brain dysfunction and structural impairment. We hypothesize that MMT plus add-on low-dose memantine will not only reduce tolerance to methadone and shorten the time for efficacious MMT, but also reduce neuronal damage caused by methadone. These proposed effects of memantine may require further data such as opioid withdrawal measures, opioid craving measures, and neurocognitive function evaluation to confirm.

We found that add-on low-dose memantine was beneficial for attenuating plasma TNF-α levels in opioid-dependent patients. However, a longer follow-up period (i.e., at least 6 months) is necessary in future experiments to confirm our finding. We also found initial evidence of significant difference in the plasma TGF-β levels in the two treatment groups, considering the difference in increase of the TGF-β levels appears to be because the levels in Placebo group dropped. The TGF-β1 is a potent anti-inflammatory cytokine that regulates various physiological processes, viz., cell proliferation, cell differentiation, and extracellular matrix synthesis, and that inhibits cellular and humoral immune responses and cytokine production^22^. Furthermore, TGF-β1 induces plasminogen activator inhibitor 1 (PAI-1) synthesis and stimulates the synthesis of collagen and α-actin in vascular smooth muscle cells^23^. Therefore, TGF-β1 has been implicated in the pathogenesis of autoimmune disease, carcinogenesis, and cardiovascular disease^24^. Evidence indicates that TGF-β is implicated in cardiovascular disease with significantly higher plasma levels of activated TGF-β in patients with coronary heart disease^25^. We hypothesize that memantine increased the activation of the immune response to heroin and methadone use by maintaining the plasma TGF-β1 levels. However, whether this is, in fact, the mechanism must be confirmed in future studies.

Studies on memantine’s therapeutic effect on opioid dependence are scarce, and those that do exist have small study populations and controversial findings. When used alone at higher doses (30 or 60 mg/day), memantine attenuated the symptoms of opioid withdrawal^26^ and modestly reduced the craving for heroin^27^. As an adjunct (30 or 60 mg/day) to oral naltrexone in a more recent clinical study, however, memantine did not increase treatment retention or mitigate the symptoms of opioid withdrawal or heroin craving^28^. Clinical studies of memantine’s therapeutic effects for other types of substance dependence—alcohol, cocaine, and nicotine—are contradictory and mostly negative^29^. Other researchers have attributed memantine’s beneficial effects against substance abuse to its NMDA blocker effect^30^,^31^. In the current study, patients were given only 5 mg of memantine per day, and their plasma memantine concentration was about 10–50 ng/ml (0.05–0.2 μM). Such a low dose of plasma memantine was not high enough to block the NMDA receptors (50% inhibition concentration [IC_50_] of memantine: 2-3 μM)^32^. We previously^33^ reported an alternative mechanism for memantine: an anti-inflammatory effect by reducing the activity of microglia and an increase in the release of neurotrophic factors by astroglia, which are mechanistically remote from an NMDA receptor. We hypothesize that the decline in TNF-α and increase in TGF-β1 levels in the current study were the result of memantine’s anti-inflammatory effect, not its function as an NMDA-receptor blocker. However, current study design did not address this mechanistic concern; additional mechanistic studies are necessary to confirm this hypothesis.

When treating neuropsychiatric disorders, being able to identify and quantify peripheral biomarkers for diagnosis or monitoring treatment response still remains a clinical goal. Some studies have suggested that changes in proinflammatory cytokines and BDNF may be related to the pathophysiology of opioid dependence^6^,^34^,^35^. In the current study, we found that add-on memantine was no more effective than was placebo for modulating IL-6, IL-8, CRP, and BDNF levels in opioid-dependent patients. Furthermore, memantine was no more effective than was placebo for increasing the retention rate in the trial. Whether memantine can improve the MMT completion rate requires further study. We suspect that 12 weeks may not be long enough to detect other clinical and immunological improvements. At least 6 months of treatment may be needed.

Our study has some limitations. First, we measured plasma cytokines because previous studies suggested that changes in peripheral cytokine secretion might indicate changes in central levels. However, like other studies (e.g.,^36^), we were unable to arrive at a definitive conclusion about this. Second, our study was undoubtedly too short and our study populations too small to confirm our positive findings. Third, the current study support that memantine may decrease does of methadone. However, reduction in dose of methadone not necessarily reflects decrease of methadone tolerance, relief of opioid withdrawal and craving for heroin. We did not use the clinical opiate withdrawal scale (COWS) to measure withdrawal symptoms. These hypotheses need be confirmed with further data collected. Furthermore, if we correct for multiple comparisons, our positive findings for memantine’s beneficial effects may not hold up. In addition, we did not explore other factors, such as smoking and weight, which could influence the effects of memantine. Finally, because the present study was a fixed-dose comparison without dose-assessment trials, the definitive effects of add-on memantine and their clinical efficacy require additional studies.

In conclusion, we found that treating opioid-dependent patients undergoing MMT with add-on memantine decreased the methadone dose required, significantly reduced plasma TNF-α levels, and significantly increased TGF-β1 levels, but that it had little effect on other cytokines. Our data support the efficacy of memantine in treating opioid-dependent patients on MMT. We conclude that low-dose memantine might be a feasible adjuvant therapy for attenuating inflammation and inhibiting methadone tolerance.

## Additional Information

**How to cite this article**: Lee, S.-Y. *et al.* Low-dose memantine attenuated methadone dose in opioid-dependent patients: a 12-week double-blind randomized controlled trial. *Sci. Rep.* **5**, 10140; doi: 10.1038/srep10140 (2015).

## Figures and Tables

**Table 1:**
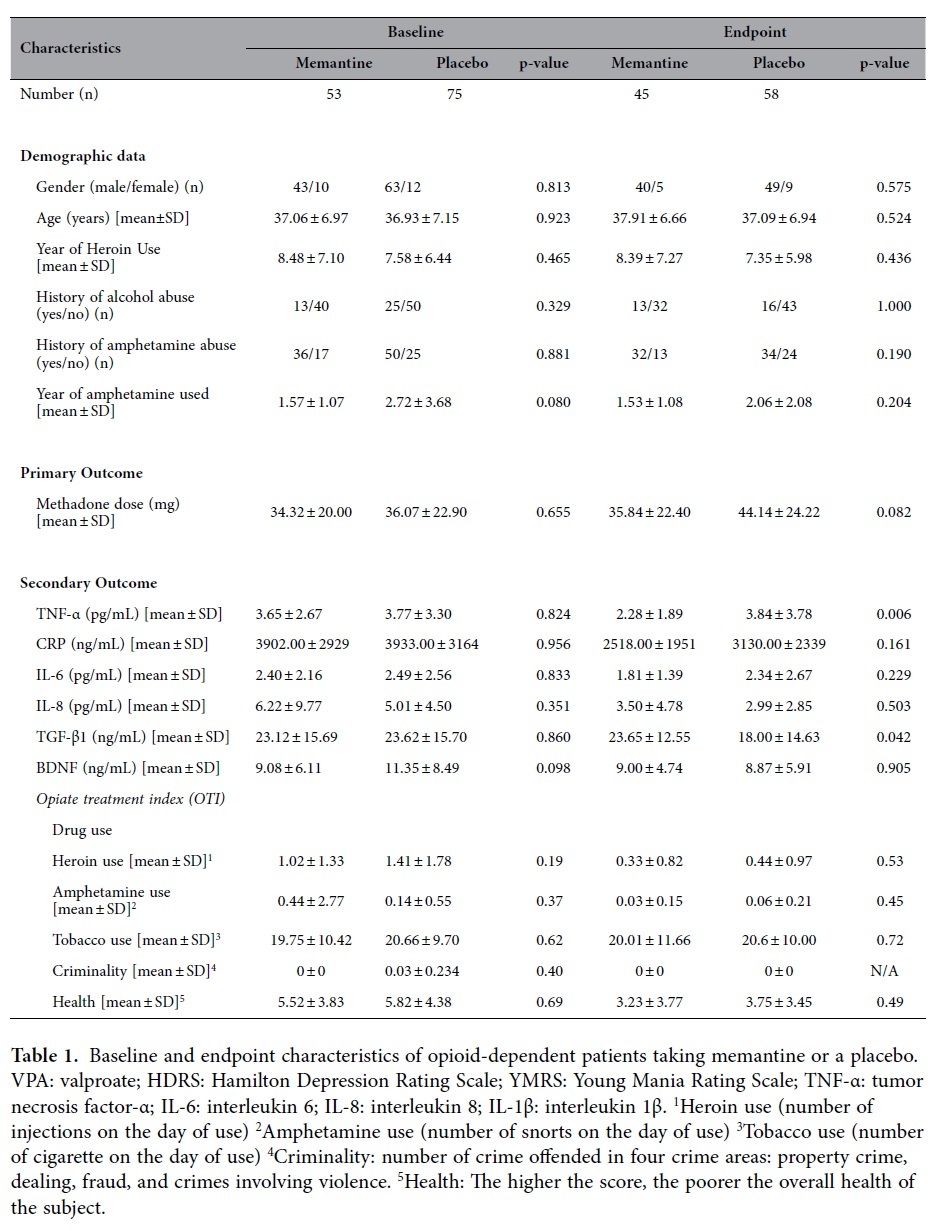
Baseline and endpoint characteristics of opioid-dependent patients taking memantine or a placebo.

**Table 2:** Effect of memantine vs. placebo during 12 weeks of treatment on primary and secondary outcomes in heroin-dependent patients.

**Parameter**	**Covariant**	**Estimate**	**SE**	***t***	**p-value**	**AIC**^1^
**Primary Outcome**						
**Methadone dose required**^**2,4**^	Treatment received X Treatment course	−0.948	0.446	−2.128	0.034*	5709.7
	Treatment received	0.135	3.957	0.034	0.973	
	Treatment course	1.307	0.291	4.488	<0.001	
	Age	−0.904	0.273	−3.303	0.001	
	Gender	−0.068	5.099	−0.013	0.989	
**% of change from baseline in methadone dose required**^**2**^	Treatment received X Treatment course	−0.031	0.014	−2.242	0.025*	
	Treatment received	0.044	0.059	0.749	0.454	497.0
	Treatment course	0.051	0.009	5.545	<0.001	
	Age	0.004	0.003	1.630	0.106	
	Gender	−0.071	0.051	−1.392	0.166	
**Secondary Outcome**						
**TNF-α (pg/mL)**	Treatment received X Treatment course	−0.035	0.012	−2.924	0.004**	300.4
	Treatment received	0.078	0.070	1.117	0.265	
	Treatment course	0.003	0.008	0.425	0.671	
	Age	0.004	0.004	1.009	0.315	
	Gender	−0.045	0.079	−0.567	0.572	
**CRP (pg/mL)**	Treatment received X Treatment course	−0.017	0.010	−1.630	0.104	228.0
	Treatment received	0.016	0.077	0.213	0.832	
	Treatment course	−0.014	0.007	−2.055	0.040	
	Age	−0.002	0.005	−0.378	0.706	
	Gender	−0.045	0.096	−0.475	0.636	
**IL-6 (pg/mL)**^**2**^	Treatment received X Treatment course	0.003	0.010	0.283	0.777	181.4
	Treatment received	−0.007	0.064	−0.114	0.909	
	Treatment course	−0.016	0.007	−2.351	0.019	
	Age	0.007	0.004	1.657	0.100	
	Gender	−0.131	0.076	−1.733	0.085	
**IL-8 (pg/mL)**	Treatment received X Treatment course	−0.016	0.017	−0.921	0.357	655.0
	Treatment received	0.092	0.091	1.014	0.312	
	Treatment course	−0.032	0.011	−2.923	0.004	
	Age	0.002	0.015	0.430	0.668	
	Gender	0.019	0.099	0.190	0.850	
**TGF-β1 (pg/mL)**	Treatment received X Treatment course	0.028	0.012	2.403	0.017*	231.7
	Treatment received	−0.057	0.058	−0.997	0.319	
	Treatment course	−0.016	0.008	−2.077	0.038	
	Age	−0.010	0.003	−3.219	0.002	
	Gender	0.114	0.059	1.915	0.058	
**BDNF (pg/mL)**	Treatment received X Treatment course	312.750	212.400	1.472	0.142	12273.6
	Treatment received	−2066.35	1168.28	−1.769	0.078	
	Treatment course	−344.76	138.88	−2.482	0.013	
	Age	−258.48	68.47	−3.775	<0.001	
	Gender	3090.11	1293.35	2.389	0.018	

SE: Standard Error; TNF-α: tumor necrosis factor-α; CRP: C-reactive protein; IL-6: interleukin 6; IL-8: interleukin 8; TGF-β1: transforming growth factor β1; BDNF: brain-derived neurotrophic factor.

Primary outcomes and secondary outcomes are dependent variables. Independent variable shown here is the interaction of treatment received and treatment course. Other covariables are treatment received, treatment course, gender, age. Reference group is Placebo group.

^*^p < 0.05,

^**^p < 0.01

^1^AIC: Akaike’s Information Criteria.

**Table 3:** Hazard ratio (HR) of dropout during the trial (n = 25) (Cox proportional hazards model).

	**Dropout rate**	**Adjusted hazard ratio model**	
**Treatment Group**	**n/Total n (%)**	**Exp (B)**	**p-value**
Memantine	8/45 (15.1)	1 (Ref)	
Placebo	17/58 (22.7)	1.586	0.282

Exp (B): odds ration; (Ref): reference group.

**Table 4:** Evaluation of Side effect Checklist at endpoint for opioid-dependent patients taking memantine or a placebo.

**Subscales from side effect checklist**	**Memantine**	**Placebo**	**p-value**
A. Mental Status [mean ± SD]	0.14 ± 0.47	0.06 ± 0.24	0.31
B. Urogenital system [mean ± SD]	0.81 ± 0.96	0.48 ± 0.85	0.07
C. Cardiovascular System [mean ± SD]	0.02 ± 0.15	0.02 ± 0.14	0.91
D. Head and neck [mean ± SD]	0.21 ± 0.47	0.31 ± 0.76	0.43
E. Four limbs [mean ± SD]	0.07 ± 0.34	0.04 ± 0.28	0.64
F. Skin [mean ± SD]	0.14 ± 0.35	0.17 ± 0.38	0.63
G. Gastrointestinal system [mean ± SD]	0.07 ± 0.34	0.04 ± 0.28	0.63

Score of symptoms: 0 = absent, 1 = mild, 2 = moderate, 3 = severe.

## References

[b1] Mattick R. P., Breen C., Kimber J., Davoli M., Buprenorphine maintenance versus placebo or methadone maintenance for opioid dependence. Cochrane Database Syst. Rev. 2, CD002207 (2014).24500948

[b2] Thomas P. T., Bhargava H. N., House R. V., Immunomodulatory effects of *in vitro* exposure to morphine and its metabolites. Pharmacology . 50, 51–62 (1995). 7899480 10.1159/000139266

[b3] Kapasi A. A., Gibbons N., Mattana J., Singhal P. C., Morphine stimulates mesangial cell TNF-alpha and nitrite production. Inflammation . 24, 463–476 (2000). 10921509 10.1023/a:1007016329300

[b4] Zubelewicz B., Muc-Wierzgon M., Harbuz M. S., Brodziak A., Central single and chronic administration of morphine stimulates corticosterone and interleukin (IL)-6 in adjuvant-induced arthritis. J. Physiol. Pharmacol. 51, 897–906 (2000). 11220497

[b5] Dyuizen I., Lamash N. E. Histo- and immunocytochemical detection of inducible NOS and TNF-alpha in the locus coeruleus of human opiate addicts. J. Chem. Neuroanat. 37, 65–70 (2009). 19038328 10.1016/j.jchemneu.2008.10.005

[b6] Angelucci F. *et al.* Chronic heroin and cocaine abuse is associated with decreased serum concentrations of the nerve growth factor and brain-derived neurotrophic factor. J. Psychopharmacol. 21, 820–825 (2007). 17715210 10.1177/0269881107078491

[b7] Graham D. L. *et al.* Dynamic BDNF activity in nucleus accumbens with cocaine use increases self-administration and relapse. Nat. Neurosci. 10, 1029–1037 (2007). 17618281 10.1038/nn1929

[b8] Bolanos C. A., Nestler E. J., Neurotrophic mechanisms in drug addiction. Neuromolecular Med. 5, 69–83 (2004). 15001814 10.1385/NMM:5:1:069

[b9] Mattick R. P., Breen C., Kimber J., Davoli M., Methadone maintenance therapy versus no opioid replacement therapy for opioid dependence. Cochrane Database Syst. Rev. 10.1002/14651858.CD002209.pub2,CD002209 (2009). 12519570

[b10] Mattick R. P., Kimber J., Breen C., Davoli M., Buprenorphine maintenance versus placebo or methadone maintenance for opioid dependence. Cochrane Database Syst. Rev. 10.1002/14651858.CD002207.pub3,CD002207 (2008). 18425880

[b11] Bruce-Keller A. J. *et al.* Morphine causes rapid increases in glial activation and neuronal injury in the striatum of inducible HIV-1 Tat transgenic mice. Glia . 56, 1414–1427 (2008). 18551626 10.1002/glia.20708 PMC2725184

[b12] Chen S. L. *et al.* Low-dose memantine attenuated morphine addictive behavior through its anti-inflammation and neurotrophic effects in rats. J. Neuroimmune Pharmacol. 7, 444–453 (2012). 22205542 10.1007/s11481-011-9337-9 PMC3611110

[b13] Lee S. Y. *et al.* The Effects of Add-On Low-Dose Memantine on Cytokine Levels in Bipolar II Depression: A 12-Week Double-Blind, Randomized Controlled Trial. J. Clin. Psychopharmacol. 34, 337–343 (2014). 24717258 10.1097/JCP.0000000000000109

[b14] Sheehan D. V. *et al.* The Mini-International Neuropsychiatric Interview (M.I.N.I.): the development and validation of a structured diagnostic psychiatric interview for DSM-IV and ICD-10. J. Clin. Psychiatry. 59 **Suppl 20**, 22–33; quiz 34-57 (1998). 9881538

[b15] Endicott J., Spitzer R. L., A diagnostic interview: the schedule for affective disorders and schizophrenia. Arch. Gen. Psychiatry. 35, 837–844 (1978). 678037 10.1001/archpsyc.1978.01770310043002

[b16] Liao D. L. *et al.* Higher methadone doses are associated with lower mortality in patients of opioid dependence in Taiwan. J. Psychiatr. Res. 47, 1530–1534 (2013). 23880602 10.1016/j.jpsychires.2013.07.001

[b17] D’Aunno T., Pollack H. A., Frimpong J. A., Wuchiett D., Evidence-based treatment for opioid disorders: a 23-year national study of methadone dose levels. J. Subst. Abuse Treat. 47, 245–250 (2014). 25012549 10.1016/j.jsat.2014.06.001 PMC4139092

[b18] Webster L. R., Reisfield G. M., Dasgupta N., Eight principles for safer opioid prescribing and cautions with benzodiazepines. Postgrad. Med. 127, 27–32 (2015). 25526233 10.1080/00325481.2015.993276

[b19] Shi J. *et al.* PET imaging of dopamine transporter and drug craving during methadone maintenance treatment and after prolonged abstinence in heroin users. Eur. J. Pharmacol. 579, 160–166 (2008). 17977528 10.1016/j.ejphar.2007.09.042

[b20] Mintzer M. Z., Stitzer M. L., Cognitive impairment in methadone maintenance patients. Drug Alcohol Depend. 67, 41–51 (2002). 12062778 10.1016/s0376-8716(02)00013-3

[b21] Prosser J., London E. D., Galynker, II, Sustained attention in patients receiving and abstinent following methadone maintenance treatment for opiate dependence: performance and neuroimaging results. Drug Alcohol Depend. 104, 228–240 (2009). 19608356 10.1016/j.drugalcdep.2009.04.022

[b22] Yang Y. C. *et al.* Transforming growth factor-beta1 in inflammatory airway disease: a key for understanding inflammation and remodeling. Allergy . 67, 1193–1202 (2012). 22913656 10.1111/j.1398-9995.2012.02880.x

[b23] Vaughan D. E., PAI-1 and TGF-beta: unmasking the real driver of TGF-beta-induced vascular pathology. Arterioscler. Thromb. Vasc. Biol. 26, 679–680 (2006). 16556860 10.1161/01.ATV.0000209949.86606.c2

[b24] Redondo S., Navarro-Dorado J., Ramajo M., Medina U., Tejerina T., The complex regulation of TGF-beta in cardiovascular disease. Vasc. Health Risk Manag. 8, 533–539 (2012). 23028232 10.2147/VHRM.S28041 PMC3446857

[b25] Lu Y. *et al.* TGFB1 genetic polymorphisms and coronary heart disease risk: a meta-analysis. BMC Med. Genet. 13, 39 (2012). 22607024 10.1186/1471-2350-13-39 PMC3497590

[b26] Bisaga A. *et al.* The NMDA antagonist memantine attenuates the expression of opioid physical dependence in humans. Psychopharmacology (Berl.) . 157, 1–10 (2001). 11512037 10.1007/s002130100739

[b27] Comer S. D., Sullivan M. A., Memantine produces modest reductions in heroin-induced subjective responses in human research volunteers. Psychopharmacology (Berl.) . 193, 235–245 (2007). 17406858 10.1007/s00213-007-0775-2

[b28] Bisaga A. *et al.* A placebo controlled trial of memantine as an adjunct to oral naltrexone for opioid dependence. Drug Alcohol Depend. 119, e23–29 (2011). 21715107 10.1016/j.drugalcdep.2011.05.019 PMC3199033

[b29] Sani G. *et al.* The role of memantine in the treatment of psychiatric disorders other than the dementias: a review of current preclinical and clinical evidence. CNS Drugs. 26, 663–690 (2012). 22784018 10.2165/11634390-000000000-00000

[b30] Muhonen L. H., Lonnqvist J., Juva K., Alho H., Double-blind, randomized comparison of memantine and escitalopram for the treatment of major depressive disorder comorbid with alcohol dependence. J. Clin. Psychiatry. 69, 392–399 (2008). 18348597 10.4088/jcp.v69n0308

[b31] Zdanys K., Tampi R. R., A systematic review of off-label uses of memantine for psychiatric disorders. Prog. Neuropsychopharmacol. Biol. Psychiatry. 32, 1362–1374 (2008). 18262702 10.1016/j.pnpbp.2008.01.008

[b32] Parsons C. G., Danysz W., Quack G., Memantine is a clinically well tolerated N-methyl-D-aspartate (NMDA) receptor antagonist—a review of preclinical data. Neuropharmacology . 38, 735–767 (1999). 10465680 10.1016/s0028-3908(99)00019-2

[b33] Wu H. M. *et al.* Novel neuroprotective mechanisms of memantine: increase in neurotrophic factor release from astroglia and anti-inflammation by preventing microglial activation. Neuropsychopharmacology . 34, 2344–2357 (2009). 19536110 10.1038/npp.2009.64 PMC3655438

[b34] Ghazavi A., Mosayebi G., Solhi H., Rafiei M., Moazzeni S. M., Serum markers of inflammation and oxidative stress in chronic opium (Taryak) smokers. Immunol. Lett. 153, 22–26 (2013). 23850638 10.1016/j.imlet.2013.07.001

[b35] Ghazavi A., Solhi H., Moazzeni S. M., Rafiei M., Mosayebi G., Cytokine profiles in long-term smokers of opium (Taryak). J. Addict. Med. 7, 200–203 (2013). 23519052 10.1097/ADM.0b013e31828baede

[b36] Pan W., Kastin A. J., Penetration of neurotrophins and cytokines across the blood-brain/blood-spinal cord barrier. Adv. Drug Deliv. Rev. 36, 291–298 (1999). 10837721 10.1016/s0169-409x(98)00086-6

